# Hospital-Based Prevalence of Iron Deficiency Anemia among Pre-School Children in Dubai

**DOI:** 10.7759/cureus.10894

**Published:** 2020-10-11

**Authors:** Wafaa Faysal, Abdul Rehman Z Zaidi, Sameer Al-Abdi, Saad Alhumaid, Maied Z AlShehery, Abbas Al Mutair

**Affiliations:** 1 Pediatrics, Dr. Sulaiman Al Habib Medical Group, Dubai, ARE; 2 Internal Medicine, Dr. Sulaiman Al Habib Medical Group, Riyadh, SAU; 3 Internal Medicine, Alfaisal University, Riyadh, SAU; 4 Pediatrics, King Abdulaziz Hospital, Ministry of the National Guard-Health Affairs, Al-Ahsa, SAU; 5 Infectious Disease, Ministry of Health, Riyadh, SAU; 6 Palliative Care, King Fahad Medical City, Riyadh, SAU; 7 Internal Medicine, University of Wollongong, Wollongong, AUS

**Keywords:** iron deficiency anemia, anaemia, iron deficiency, united arab emirates, dubai, prevalence, children, pediatrics, anemia, ida

## Abstract

Introduction: Iron deficiency anemia (IDA) is an internationally recognized leading cause of disability and contributes to childhood morbidity and mortality. The prevalence of IDA is higher in developing countries, especially in Arab countries, compared to the west.

Methods: To assess the prevalence of IDA, we analyzed the data of children aged between one to five years seen at Dr. Sulaiman Al-Habib Medical Group’s tertiary care hospital in Dubai, United Arab Emirates (UAE) from 2016 to 2018.

Results: We found a high occurrence of IDA in male children and non-Emirati children.

Conclusion: Appropriate screening and iron supplementation are required to see a decline in the rate of IDA. Further nationwide studies are required to identify the highly prevalent and high-risk areas of IDA in the UAE.

## Introduction

Anemia can be defined as a decreased count of hemoglobin, red blood cells, and with possible alteration of red blood cell morphology [1]. There are many types of anemias and they are driven by multiple factors. The most common type of anemia is iron deficiency anemia (IDA) which affects more than 25% of the world’s population [2]. IDA is the third leading cause of disability worldwide and contributes to childhood morbidity and mortality. According to a report by the World Health Organization, the global burden of IDA in 2000 was 1946.4 years of life lost, 9430.1 years lost due to disability, and 11376.5 disability-adjusted life years [3].

Iron is important for the development of the central nervous system, especially during the first two years of life, hence a deficiency can cause cognitive decline [4]. IDA is a diversely distributed disease that mainly affects children, female teenagers, pregnant and lactating women [5]. Rapidly growing children (from 0-15 years of age) may consume iron stores that accrue during gestation, which can physiologically lead to an absolute deficiency in ferritin, making them one of the highest risk prone population of contracting IDA [6]. IDA can also be associated with low birth weight and a higher risk of maternal and perinatal mortality [7]. Symptoms occur due to reduced oxygen delivery to body tissues and may include dyspnea, headache, lethargy, paleness, difficulty in concentration, and can affect motor and mental development [8]. Severe IDA can also increase the risk of premature delivery, low birth weight, mortality during labor, higher rates of infections, and cardiac failure [9,10].

The prevalence of iron deficiency seems to be higher in resource-deprived and developing countries, especially in infants in Arab countries (~72%), compared to developed nations [11-13]. The incidence rate of IDA in infants in a suburb of the United Arab Emirates (UAE) was previously reported to be ~23.2% [14]. As data from our region is scarce, we investigated the prevalence and predictors of IDA in children aged one to five years.

## Materials and methods

We retrospectively reviewed records of 1595 preschooler children (1-5 years old) who were seen at Dr. Sulaiman Al-Habib Medical Group’s private tertiary care hospital in Dubai, UAE from 2016 to 2018 to assess the prevalence of IDA.

We recorded the nationality of each study children and later we dichotomized the nationality as Emirati and Non-Emirati for analysis. We extracted white blood cell (WBC) count, hemoglobin level, ferritin level, iron supplementation, age, and gender of each study children. We defined IDA according to the criteria of the Centers for Disease Control and Prevention; a hemoglobin concentration <11 g/dl and serum ferritin <10 µg/L [15,16]. As ferritin may increase in an infectious or inflammatory state, we excluded any children that had WBC >10 x 109/L as a surrogate for any possible infections. We also recorded information on which of the children were already on iron supplements. The Institutional Review Board approved the study without a need to obtain written consent from the parents (IRB Log No. RC19.04.35).

We divided the study sample into two groups. In group 1, we wanted to test if a hemoglobin level <11 with lower than normal mean corpuscular volume (MCV) and/or high red cell distribution width (RDW) can identify IDA alone. Similarly, if ferritin of <20 can alone identify IDA correctly with a condition that WBCs were not >10,000. In group 2, hemoglobin <11 and ferritin <20 together were used to predict IDA more accurately.

Data were analyzed using IBM Statistical Package for the Social Sciences (SPSS) version 24 (SPSS Inc, Chicago, IL). A value of p <0.05 was considered statistically significant. Our response variable is IDA that is classified into whether iron deficiency is present or not. Since our response was binary, we performed binary logistic regression. We applied binary logistic regression taking the predictors as gender, nationality, supplement, and age. Moreover, gender and nationality were our categorical variables. After executing binary logistic regression, we obtained the odds ratio and p-value of each predictor/factor. The model estimates are presented with the adjusted odds ratios and 95% confidence interval (CI).

## Results

**Figure 1:**
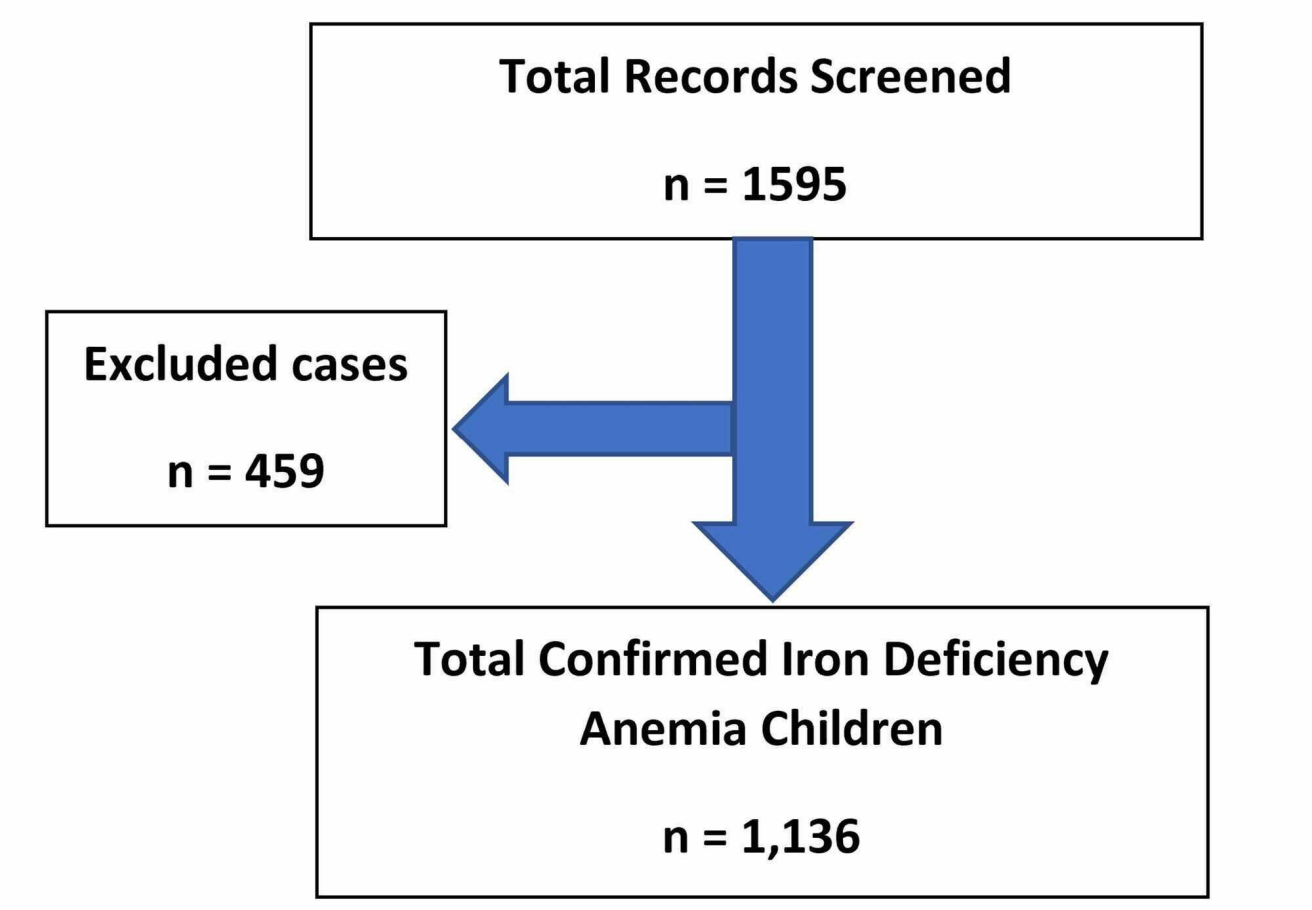
Exclusion of cases with white blood cell (WBC) >10,000 x 10^9/L

As seen in Table 1, among 1595 children, 800 (~50%) belonged to group 1, among which 444 (55.5%) were male and 356 (44.5%) were female. 224 children belonged to group 2 which were 14% of the sample having both hemoglobin and ferritin less than 11 and 20, respectively. In this category, 15% were male and 12% were female. 22% of our total sample were Emirati children, 53% of them were classified into group 1 and 12% were categorized into group 2. A total of 39% of the children were taking IDA supplements. 46% of the total children were less than two years old. 48% of the total sample belonged to group 1 and 19% fell under group 2. To overcome any bias in our sample and to improve the accuracy of the study, we used WBC as an exclusion variable, and those that were having a WBC count of >10,000 were excluded. We only included 1,136 cases (71% of the total sample) in the study, who had a confirmed diagnosis of IDA in a tertiary care hospital setting (Figure 1). The age ranged from one to five years. Overall, more male children were affected by IDA than females.

**Table 1:** Count and percentages of factors of Hb <11 or ferritin <20 and Hb <11 and ferritin <20 Hb: hemoglobin; WBC: white blood cell; IDA: iron deficiency anemia.

Factors		Group 1: Hb <11 or Ferritin <20	Group 2: Hb <11 & Ferritin <20
Gender	Male (878)	444 (51%)	135 (15%)
	Female (717)	356 (50%)	89 (12%)
Nationality	Emirati (355)	189 (53%)	64 (18%)
	Non-Emirati (1240)	610 (49%)	160 (13%)
WBC	<10,000 (1136)	558 (49%)	196 (12%)
	>10,000 (459)	242 (15%)	28 (2%)
IDA Supplement	Yes (619)	619 (100%)	43 (7%)
	No (976)	52 (5%)	52 (5%)
Age	1 - <2 (735)	355 (48%)	143 (19%)
	2 - 3 (317)	147 (46%)	29 (9%)
	3 - 4 (320)	162 (51%)	35 (11%)
	4 - 5 (223)	136 (61%)	17 (8%)

The results of the binary logistic regression analysis can be seen in Table 2. We can see that 57% of males had IDA and 58% were in the non-IDA group. If we compare Emirati children with Non-Emirati children, 24% had IDA. There was no difference in the confidence intervals of children taking a supplement or not. The gender odds ratio (OR) was 0.977 with a p-value of 0.869 which means males were 3% more likely to have IDA, but gender is insignificant. Nationality OR was 1.539 with a significant p-value, which means Emirati children were 54% less likely to have IDA in contrast to non-Emirati children. Supplement OR was 0.872 which means those that were taking supplements had a 12.8% more chance of having IDA, but supplements are insignificant at a 5% level of significance. Age OR is 1.001 showing that age did not affect the prevalence of IDA. 

**Table 2:** Odds ratio (OR) and confidence interval (CI) of iron deficiency anemia (IDA) and non-iron deficiency anemia

Factors		IDA Count	IDA 95% CI		Non-IDA Count	Non-IDA 95% CI		OR	P-value
			Hemoglobin	Ferritin		Hemoglobin	Ferritin		
Gender	Male	350 (57%)	11.30-11.93	17.91-28.84	305 (58%)	12.11-12.29	39.87-44.97	0.977	0.869
	Female	260 (43%)	11.15-11.70	20.07-27.68	221 (42%)	12.08-12.31	46.75-61.40		
Nationality	Emirati	146 (24%)	10.97-11.50	19.05-36.29	109 (21%)	11.90-12.23	40.12-59.01	1.539	<0.01
	Non-Emirati	454 (76%)	11.35-11.91	18.69-23.46	417 (79%)	12.16-12.31	43.07-50.14		
Supplement	Yes	330 (54%)	11.27-11.71	17.66-24.56	271 (52%)	12.11-12.32	41.81-50.92	0.872	0.321
	No	280 (46%)	11.37-11.75	16.59-23.10	255 (48%)	12.09-12.27	43.03-53.19		
Age		610 (100%)	11.32-11.75	19.91-25.50	526 (100%)	12.16-12.44	43.06-48.93	1.001	0.795

## Discussion

Globally, iron deficiency is the most common nutritional disease and the root cause of anemia in children and pregnant women [17]. IDA commonly accompanies chronic illnesses such as chronic kidney disease, malignancy, rheumatoid arthritis, obesity, and inflammatory bowel disease [18-20]. The study of the prevalence of anemia, in general, is difficult as it requires an understanding of the epidemiology of the underlying causes of anemia. Therefore it is a good idea to study specific types of anemia, such as IDA, and also different severities of anemia [21]. Most studies from around the world focus on IDA due to its clinical significance and high prevalence. High-risk populations may have a total anemia occurrence of 50%-80%, and this may be due to a substandard socioeconomic status, lower body weight, and is found to be higher in new mothers [22].

In our study, we used high WBC as a surrogate marker for possible infection or acute phase reactive state that might lead to ferritin leak from the liver, falsely elevating ferritin levels, and missing the true iron deficiency state [23]. We utilized hemoglobin and ferritin levels, as ferritin is found to be a more accurate marker of IDA than traditional red cell indices, which in IDA, usually indicate that the red blood cells are microcytic and hypochromic. We found 1,024 children, with ages ranging from one-five years, to have IDA, which demonstrates a high prevalence of IDA in children in the UAE. Our findings are similar to studies done in Saudi Arabia [24,25], but show a lower incidence of IDA than a study done in Jordan [12]. The mean age of children with IDA was a year higher in our study than the mean age of 15.4 months in Saudi Arabia [24]. As previously reported in the Eastern Mediterranean region, we also observed a slightly higher incidence of IDA in male children [24,26].

We found a higher rate of IDA in non-Emirati children, and this may be because of the population of the Emiratis is lower than the expatriates living in the UAE. In 2011, it was reported that less than 1 million out of the 8.3 million residents of the UAE were Emirati [27]. We also observed the children on iron supplements had more odds of having IDA, and this might be because our study was of a retrospective design, and the children might have been already identified as IDA and those children may have been taking supplements after diagnosis of IDA.

Routine screening for anemia at 12 months of age is vital as recommended by the American Academy of Pediatrics and the World Health Organization [28]. Serum ferritin levels also need to be measured during screening to identify IDA, which if supplemented with dietary iron early, can potentially prevent cognitive defects. However, we must be cautious that the serum ferritin level is not the sole basis of diagnosis and that the child does not have an infection or any inflammatory disorder. Patients and physicians should be wary of children with poor growth, feeding problems, and inadequate dietary iron intake, as these children will need to be screened for IDA as well [29]. Routine screening for IDA is especially crucial for children living in areas with a high prevalence of IDA. This is also why national epidemiological studies to identify these regions are imperative.

To overcome iron deficiency in children, it is also essential to optimize their nutrition with targeted daily iron supplementation. Full-term breastfed infants can be started on elemental iron 1 mg/kg daily at four months of age and this can be sustained until the infant is on adequate iron-rich foods, such as meat, fortified food, and green vegetables. Such complementary iron-rich foods can be taken by six months of age [30]. Appropriate iron supplementation in children is vital in high-risk and highly prevalent areas of IDA. For children from the age of one to three years, the recommended dietary allowance for iron is 7 mg/day [30].

Our study had several limitations. Firstly, the cross-sectional nature of the study means that the chronological relationship between IDA and the associated factors cannot be established. We also could not include red cell indices of the children, which could have given us a better insight into the red blood cell morphology of the children. We also could not obtain the maternal red cell indices, nor their hemoglobin and ferritin levels to assess if the mothers also had IDA. Furthermore, we were unable to measure serum vitamin B12 and folate levels in these children and so could not quantify other forms of anemia, signifying some misclassification bias. 

## Conclusions

IDA is a serious concern and affects children worldwide, including the UAE. More children that were less than two years of age, non-Emirati, and males were affected by IDA in our findings. Rigorous screening and iron supplementation programs are necessary to see a significant decrease in this ailment. Further collaborative national studies are required to identify the high-risk and high-prevalence areas of IDA in different parts of the world.
